# The Comparative Effects of High-Intensity Interval Training and Traditional Resistance Training on Hormonal Responses in Young Women: A 10-Week Intervention Study

**DOI:** 10.3390/sports13030067

**Published:** 2025-02-25

**Authors:** Wael Ramadan, Chrysovalantou E. Xirouchaki, Abdel-Hady El-Gilany

**Affiliations:** 1 Department of Sports Training, Faculty of Physical Education, Mansoura University, Mansoura 35516, Egypt; 2 Department of Biochemistry and Molecular Biology, Monash University, Clayton, VIC 3800, Australia; chrisa.xirouchaki@monash.edu; 3 Department of Public Health Faculty of Medicine, Mansoura University, Mansoura 35516, Egypt; ahgilany@gmail.com

**Keywords:** high-intensity interval training, traditional resistance training, physical fitness, hormonal responses

## Abstract

Background: Hormonal levels in women are influenced by exercise intensity and modality. Methods: This 10-week study compared high-intensity interval training (HIIT) and traditional resistance training (TRT) in 72 young women. Hormonal levels (estrogen, testosterone, FSH, prolactin, and LH) were measured pre- and post-intervention. Results: Both groups showed significant increases in estrogen (HIIT: 150%; TRT: 72.3%) and decreases in testosterone (HIIT: 58%; TRT: 49%), FSH (HIIT: 6%; TRT: 7.7%), and PL (HIIT: 5%; TRT: 2.1%). There are no significant changes in LH. Conclusions: HIIT and TRT effectively modulate hormonal profiles, potentially benefiting reproductive and metabolic health.

## 1. Introduction

Exercise training is widely recognized for its significant benefits to young women, including notable reductions in body fat and improvements in cardiovascular health [1,2,3]. Regular exercise has crucial effects on the hormonal profile, influencing key hormones such as testosterone, estrogen, prolactin, and follicle-stimulating hormone (FSH). These hormonal changes play critical roles in various physiological functions, including reproductive health, metabolism, and overall well-being. Testosterone, primarily known for its role in muscle growth and maintenance, is significantly influenced by exercise, particularly through traditional resistance training (TRT). Studies have consistently shown that TRT exercises, like weightlifting, increase testosterone levels in both men and women, promoting muscle hypertrophy and strength [4].

Estrogen is another hormone significantly influenced by exercise, with its effects varying depending on factors such as exercise intensity, duration, and individual physiological characteristics. The relationship between exercise and estrogen regulation is complex and may differ across populations and activity types. Endurance exercise, like long-distance running or swimming, is associated with reductions in estrogen levels in women, possibly due to changes in body fat composition and hormonal regulation [5,6]. Prolactin, a hormone crucial for lactation and reproductive function, also responds to exercise. Moderate- to high-intensity exercise can transiently increase prolactin levels, particularly in women, reflecting the body’s response to physical stress and metabolic demands [7]. Follicle-stimulating hormone (FSH), involved in reproductive health and the regulation of the menstrual cycle, may exhibit altered patterns with exercise, especially in women engaged in intense training or endurance activities. Changes in FSH levels can influence menstrual regularity and fertility, highlighting exercise as a factor in reproductive health management. Exercise plays a critical role in regulating hormonal dynamics, which are essential for maintaining overall physiological homeostasis. A comprehensive understanding of these effects is crucial for optimizing exercise interventions across different populations. This knowledge can inform the development of evidence-based exercise protocols for athletes, individuals seeking to enhance reproductive health, and those managing endocrine disorders [8].

High-intensity interval training (HIIT) exerts a profound influence on hormonal regulation, affecting key endocrine markers such as testosterone, estrogen, follicle-stimulating hormone (FSH), and prolactin. This training modality, characterized by repeated cycles of high-intensity exercise interspersed with recovery periods, has been shown to significantly elevate testosterone levels in both men and women. These hormonal adaptations contribute to enhanced muscle hypertrophy, improved metabolic efficiency, and optimized energy utilization, ultimately supporting physiological adaptations to exercise. [9,10]. Conversely, immediately post-exercise, it may transiently suppress estrogen in women, potentially influencing reproductive health and bone metabolism [11]. FSH, crucial for regulating ovarian function, may exhibit altered patterns in response to HIIT, affecting menstrual regularity and fertility [12]. Prolactin, a hormone primarily involved in lactation and reproductive function, exhibits a transient increase during and after high-intensity interval training (HIIT). This acute elevation is indicative of the body’s neuroendocrine response to exercise-induced stress and the heightened metabolic demands associated with high-intensity exertion. The modulation of prolactin levels in response to HIIT underscores the complex interplay between exercise intensity, hypothalamic–pituitary axis activation, and endocrine regulation, with potential implications for performance optimization, recovery processes, and overall hormonal homeostasis [13].

Although exercise training has many physiological benefits, a potential adverse effect is primary or secondary amenorrhea, which may be precipitated by factors such as inadequate nutrition or low body mass. Both primary and secondary amenorrhea are characterized by the suppression of luteinizing and follicle-stimulating hormones. Furthermore, these conditions may involve the hypothalamic–pituitary–gonadal and hypothalamic–pituitary–adrenal axes [5].

It is worth highlighting that hypoestrogenism can adversely influence bone growth in athletes. Symptoms such as osteopenia, amenorrhea, and eating disorders are indicative of fatigue in female athletes. Girls who experience delayed menarche are more likely to have osteopenia, a higher prevalence of scoliosis, and increased stress characteristics compared to girls with typical menarche [14,15]. To maintain hormonal blood levels, it is necessary to achieve a balance between metabolism, production, and clearance rates. Various factors can disrupt this equilibrium, including vigorous physical activities, heat generation, competition-related stress, dietary restrictions, a decrease in body fat mass, and hypoxia [16]. Furthermore, consistent engagement in high-intensity exercise can result in menstrual irregularities, specifically oligomenorrhea, and can cause a disruption in the production of gonadotropins, leading to luteal phase deficiency and anovulation [17].

Prior research has shown that high-intensity and prolonged training can negatively impact female reproductive hormones. Evidence indicates that intense exercise can lead to disruptions in the circulation of female reproductive hormones and the hypothalamus–ovarian axis. These disruptions can be attributed to increased concentrations of stress hormones, elevated leptin levels, and fatigue associated with low energy availability. This disruption could result in irregular menstrual cycles related to anovulation deficiency and luteal phase.

Recently, there has been attention on the patterns of exercise, particularly examining HIIT, which involves alternating vigorous exercise with lower-intensity exercise. Compared to more traditional continuous exercise, many studies have shown that HIIT holds promise for improving cardiovascular health-related factors such as insulin resistance, glucose uptake, vascular function, body adiposity, and blood pressure [18]. Additionally, there is evidence that combining aerobic and TRT exercise may be superior in improving the control of metabolic syndrome [19]. Despite extensive research on exercise-induced hormonal changes, few studies directly compare HIIT and TRT, particularly in healthy young women. Understanding these differences can help to optimize exercise interventions for hormonal health. These studies demonstrate the need for further examination of both the pattern of exercise and the combination of exercise modalities on the hormonal profile of young women, which is why we conducted the current study. This study aims to compare the effects of HIIT and TRT on levels of female hormones, including estrogen, testosterone, LH, FSH, and prolactin.

## 2. Materials and Methods

### 2.1. Participants

This study was designed as an intervention comparative study. Seventy-two healthy, physically active female college students were recruited for this study and randomly assigned to one of two experimental groups: high-intensity power training (HIPT) (*n* = 36) or traditional resistance training (TRT) (*n* = 36). The study followed a randomized controlled trial (RCT) design to minimize selection bias and enhance the validity of the findings. Participants were screened based on predefined exclusion criteria, which included pregnancy, smoking, a diagnosis of Type II diabetes, acute or chronic cardiovascular disorders, abnormal menstrual function (e.g., amenorrhea or irregular menstruation), contraindications to physical activity, and the use of hormonal supplementation [20]. All participants provided both oral and written informed consent after receiving a comprehensive explanation of the study’s objectives, methodology, potential risks, and safety precautions. The study protocol was reviewed and approved by the Institutional Review Board (IRB) of the Faculty of Physical Education, Mansoura University, with the assigned ethics code 1175. The study was conducted in full compliance with the ethical principles outlined in the Declaration of Helsinki [21].

### 2.2. Study Design

Following the provision of informed consent, participants received detailed instructions on the movements included in the training protocol, as well as the procedures for performance assessments to ensure their safety and the proper execution of the exercises. Prior to the intervention, each participant underwent a comprehensive strength assessment to determine their maximal muscular strength for each movement prescribed in the training protocol, conducted on a designated testing day.

The intervention consisted of a 10-week structured training program, with sessions scheduled three times per week, resulting in a total of 30 training sessions. A minimum recovery period of 24 h was mandated between consecutive sessions to facilitate adequate physiological recovery. Participants underwent performance assessments both before and after the 10-week intervention to evaluate training-induced adaptations.

To monitor physical activity levels and training adherence, participants maintained a daily exercise log, documenting the duration, intensity, and type of exercise performed, along with their ratings of perceived exertion (RPE), subjective feedback regarding their physiological responses to the training, and any relevant observations regarding their general health [22].

### 2.3. HIPT Protocol

Participants were randomly assigned to one of two interventions: a standardized HIIT aerobic exercise training regimen combined with TRT, or a stretching exercise program. The program was designed to progressively reach the target volumes of 150–300 min of weekly exercise recommended for weight management [20]. Participants were instructed to engage in the prescribed exercise sessions on more than 5 days per week. Trained physical educators scheduled a weekly motivational call with the participants to assess intervention adherence, address barriers to exercise, and provide motivational counseling. Initially, all participants completed a 20 min HIIT exercise protocol consisting of alternating 2 min of brisk walking followed by 2 min of easy walking. Each week, the total exercise time increased by 5 min (to reach a total of 50 min of daily exercise), as tolerated, and the intervals of brisk walking increased in duration by 1 min until the participant could complete 5 min of brisk walking (followed by 2 min of easy walking). Participants had the option to complete the HIIT exercise in one or two sessions daily to reach the prescribed volume and pattern of exercise. The intensity of HIIT was set at 75–90% of maximum heart rate during the program. Throughout all exercise training sessions, participants were instructed to wear Polar watches to monitor their heart rates.

### 2.4. TRT Protocol

Participants assigned to the HIIT plus TRT intervention also completed a series of TRT exercises targeting the major muscle groups for 2 days a week for approximately 30 min. The exercises involved using elastic bands, light weights, and adapted bodyweight exercises. The program followed recommendations for TRT in healthy female individuals, and the intensity (resistance) of the exercises increased weekly, as tolerated [20]. The intensity of the weights used was approximately 60–80% of the one-rep max for the targeted number of repetitions.

### 2.5. Blood Samples

All blood samples were drawn by trained laboratory technicians under standardized laboratory conditions. The samples were then frozen, and assays using standardized methodologies were performed in one batch at the end of the study. Participants were asked to fast for a minimum of four hours before the blood samples were taken. The blood samples were drawn on the third day of the menstrual cycle. After collection, the samples were left to clot at room temperature for an hour and then centrifuged at 8000 rpm for 15 min at 4 °C. The obtained serum samples were stored at −20 °C until assay. Technicians separated clear, non-hemolyzed sera for use in assays of estrogen, testosterone, follicle-stimulating hormone (FSH), and luteinizing hormone (LH).

We measured hormonal concentrations using a solid-phase competitive chemiluminescent enzyme immunoassay, performed with the IMMULITE analyzer (Diagnostic Products Corporation, Los Angeles, CA. USA). This method was chosen for its established reliability, characterized by high sensitivity, specificity, and reproducibility in detecting circulating hormone levels. Venous blood samples were collected at two distinct time points: the baseline measurement, obtained prior to the initiation of the training protocol, and the post-intervention measurement, taken immediately following the final exercise session. This sampling strategy was designed to precisely evaluate the hormonal adaptations induced by the training intervention.

### 2.6. Data Analysis

Data were analyzed using the Statistical Package for the Social Sciences, version 27 (IBM Corp. Released 2020. IBM SPSS Statistics for Windows, Version 27.0. Armonk, NY, USA: IBM Corp). Quantitative variables were tested for normality using the Shapiro test and were found to have a non-parametric distribution. These variables were presented as median (minimum–maximum). The Mann–Whitney test was used for comparisons between the two groups, and the Wilcoxon Signed Ranks Test was used for paired (pre–post) comparisons within each group. To study the effect of the interaction between groups and the phase of the study (pre–post), we applied the Non-Parametric Equivalent: Aligned Rank Transform (ART), which is a non-parametric method that can handle interactions in factorial designs. It allows for the analysis of interaction effects and main effects in non-parametric data that violate ANOVA assumptions using the R program. A *p*-value of ≤0.05 was considered statistically significant.

## 3. Results

The outcomes of the current experimental procedure are displayed, and the two groups are matched, with no significant differences between them regarding age, height, body mass, and BMI (Table 1).

Table 2 shows that the two groups are matched in all hormone levels before training. Post-training, the levels of estrogen are significantly higher (45 ± 33 vs. 112.5 ± 41, and 65 ± 33 vs. 112 ± 42) with a *p*-value < 0.001 from pre- to post-training in the HIIT and TRT groups, respectively. Meanwhile, we found that testosterone levels are significantly lower (0.65 ± 0.18 vs. 0.27 ± 0.12, and 0.51 ± 0.44 vs. 0.26 ± 0.13) from pre- to post-training in the HIIT and TRT groups, respectively. The two groups showed significant increases in estrogen (HIIT: 150%; TRT: 72.3%) and decreases in testosterone (HIIT: 58%; TRT: 49%), FSH (HIIT: 6%; TRT: 7.7%), and PL (HIIT: 5%; TRT: 2.1%). There is no significant change in LH between the two groups.

**Figure 1:**
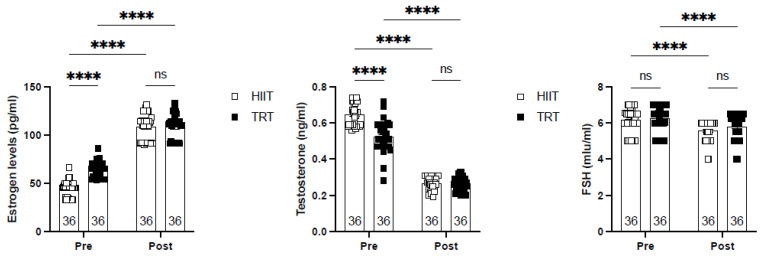
The concentrations of estrogen (pg/mL), testosterone (ng/mL), and follicle-stimulating hormone (FSH) (mIU/mL) under conditions before and after exercise training. **** (*p* < 0.0001), ns (non-significant).

Figure 1 illustrates the principal data from the present study, indicating that the 10-week training regimen considerably influenced levels of estrogen, testosterone, and follicle-stimulating hormone (FSH). Both cohorts exhibited substantial elevations in estrogen levels (HIIT: 150%; TRT: 72.3%) and reductions in testosterone (HIIT: 58%; TRT: 49%) and FSH (HIIT: 6%; TRT: 7.7%).

**Figure 2:**
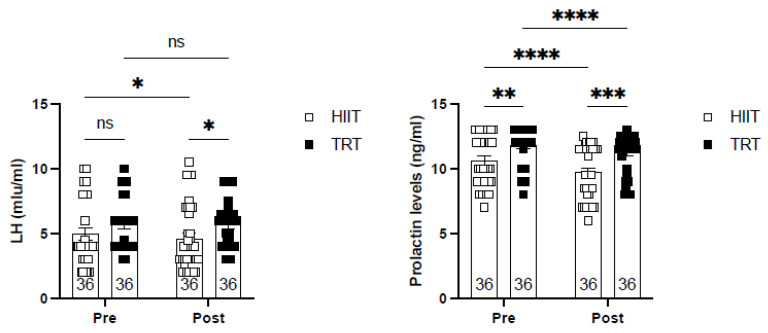
The concentrations of luteinizing hormone (LH) and prolactin (ng/mL) under conditions before and after exercise training. * (*p* < 0.05), ** (*p* < 0.01), *** (*p* < 0.001), **** (*p* < 0.0001), and ns (non-significant).

Figure 2 illustrates the concentrations of luteinizing hormone (LH) and prolactin (ng/mL) before and after exercise training. Both experimental groups exhibited significant reductions in prolactin levels, with the HIIT group showing a 5% decrease and the TRT group a 2.1% decrease. No significant alterations were observed in luteinizing hormone levels.

## 4. Discussion

Estrogen levels and other sex hormones in women are influenced by various factors, including exercise intensity, duration, menstrual phase, hormonal status, and the timing of post-exercise measurements [5,23]. These variables contribute to the inconsistencies observed in the literature regarding the specific effects of exercise on hormonal profiles. To elucidate the impact of high-intensity interval training (HIIT) on the hormonal profiles of young female individuals, we conducted the present study.

Our study demonstrated a significant increase in estrogen levels, a crucial hormone for the development and functionality of female secondary sexual characteristics and reproductive organs [24]. These findings align with those of Hackney and Willett (2020) [15], who examined the hormonal responses of young women to moderate bicycle ergometer training over eight weeks at a fixed relative intensity. Similarly, Copeland (2002) [25] carried out a study examining the hormonal responses to endurance and resistance exercise in females aged 19 to 69 years. The study found that acute bouts of both endurance and resistance exercise led to significant increases in anabolic hormones, including testosterone, estradiol, and growth hormone. Additionally, dehydroepiandrosterone (DHEA) levels increased significantly following resistance exercise. These findings suggest that exercise can acutely elevate concentrations of anabolic hormones in women across a wide age range [25]. Mannerkorpi et al. (2017) [26] emphasized the effect of physical activity on female hormonal circulation, noting increased estradiol levels following endurance and resistance exercises.

A key finding of our study was the statistically significant increase in estrogen and a significant decrease in testosterone levels. This result contrasts with Gharahdaghi et al. (2021) [14], who reported only a transient elevation in testosterone levels following resistance exercise in young, healthy females. However, a direct comparison of these results is challenging due to differences in exercise stimuli.

Kochańska-Dziurowicz et al. (2001) [24] found that acute exercise on a cycle ergometer led to a significant increase in testosterone levels in young female runners, which returned to baseline 90 min post-exercise. This underscores the importance of timing in determining testosterone levels after exercise.

Conversely, several studies have indicated that exercise does not impact testosterone levels or menstrual cycles in trained young women, suggesting that training status influences hormonal responses. Significant changes in testosterone levels have primarily been observed with resistance training interventions [27]. This indicates the necessity of different exercise stimuli and extended training durations to induce notable adaptations in testosterone levels. Further research with varied exercise stimuli and longer training periods is needed.

The existing literature suggests that physical activity generally benefits female hormonal profiles, with sex hormone levels often being used to gauge the effectiveness of physical training and to determine appropriate exercise dosages for women. Our study showed that HIIT significantly affected estrogen, testosterone, FSH, and prolactin serum hormone levels in young women, though no significant changes were observed in prolactin (PRL) and luteinizing hormone (LH) levels. The differences observed had large effect sizes (Cohen’s d ≥ 0.8), indicating practical significance.

Despite the observed increase in estrogen levels, there were no corresponding changes in serum PRL and LH levels. This result, along with the decrease in testosterone, may align with findings from a recent meta-analysis by Ennour-Idrissi et al. (2015) [5], who reported a decrease in total and free estradiol, as well as testosterone, following exercise training, particularly in non-obese individuals and after high-intensity exercise. This decrease was not fully explained by weight loss, highlighting the beneficial role of physical activity for women.

Our analysis indicates that the impact of physical activity on female sex hormones might be relatively minor and not clinically significant. Additionally, measuring sex hormone levels in the blood may not accurately reflect their effects on specific tissues. Physical activity might influence hormone function by altering the sensitivity of target tissues [28]. Estrogen secretion is regulated through a complex feedback system involving the hypothalamus, pituitary gland, and ovaries. Gonadotropin-releasing hormone (GnRH) from the hypothalamus stimulates the pituitary to release FSH and LH, which, in turn, stimulate ovarian follicles to produce estrogen. Elevated estrogen levels then inhibit GnRH and FSH release through negative feedback to regulate estrogen production [29]. Our study found increased estrogen levels despite decreased FSH after 11 weeks of HIIT in young females.

Generally, exercise tends to reduce estrogen levels, while estrogen metabolites remain unchanged [30]. However, the relationship between physical exercise and hormone levels in women remains complex. Regular exercise has been associated with reduced circulating estrogen levels [31]. It is important to consider that factors such as under-recovery and inadequate nutritional intake may also significantly affect hormone regulation. Moderate-intensity resistance training and cardiovascular exercise are linked to beneficial effects on hormones, including testosterone and progesterone, while intense exercise without proper recovery and nutrition can lead to reduced estrogen levels. A low-protein diet is associated with hormonal imbalances, including low estrogen levels [32].

## 5. Conclusions

This study demonstrates that HIIT and TRT can lead to alterations in the levels of reproductive hormones in females. Post-training assessments indicated a significant increase in estrogen levels, with values of (45 ± 33 vs. 112.5 ± 41) (pg/mL) in the HIIT group, and (65 ± 33 vs. 112 ± 42) (pg/mL) in the TRT group, respectively (*p*-value < 0.001). Conversely, testosterone levels demonstrated a significant decrease from pre- to post-training, recording values of (0.65 ± 0.18 vs. 0.27 ± 0.12) (ng/mL) in the HIIT group, and (0.51 ± 0.44 vs. 0.26 ± 0.13) (ng/mL) in the TRT group, respectively. Consequently, individuals seeking enhancements in overall fitness should consider incorporating HIIT and TRT into their training regimens. The clinical implication is that both HIIT and TRT are effective in modulating key hormonal levels, with potential applications in fitness programming for young women.

Given the hormonal adaptations described above, it is advisable for fitness professionals to incorporate HIIT and TRT protocols into training programs for female clients, with careful monitoring of individual responses to optimize endocrine health. Additionally, considering the potential impact of the menstrual cycle on training outcomes, aligning exercise regimens with different phases may enhance performance and recovery [32]. 

Future research should investigate long-term hormonal responses to combined HIIT and TRT protocols and their effects on menstrual and reproductive health, as well as metabolic outcomes.

## Figures and Tables

**Table 1:** Age and anthropometry of two groups.

	HIIT Median (Min–Max)	TRT Median (Min–Max)	Mann–Whitney Test (Independent Groups)
Age (years)	18 (17–19)	18 (17–19)	*p* ≤ 0.82
Height (cm)	167 (140–189)	166 (140–170)	*p* ≤ 0.22
Body mass (kg)	65 (40–86)	65 (40–70)	*p* ≤ 0.45
BMI (kg/m^2^)	23.3 (20.4–24.3)	23.3 (20.4–24.2)	*p* ≤ 0.76

HIIT—High-Intensity Interval Training; TRT—Traditional Resistance Training; BMI—Body mass index.

**Table 2:** Hormones of 2 groups pre- and post-intervention.

	HIIT Median (Min–Max)	TRT Median (Min–Max)	Mann–Whitney (Independent Groups)	Aligned Rank Transform (ART)
Estrogen: Pre Post % change Significance (paired) *	45 (33–66) 112.5 (90–131) +150.0 *p* ≤ 0.001	65 (53–86) 112 (91–133) +72.3 *p* ≤ 0.001	*p* ≤ 0.84 *p* ≤ 0.001	Group Effect: F (1, 70) = 13.67, *p* < 0.001 Time Effect: F (1, 70) = 2722.47, *p* < 0.001 Group × Time Interaction: F (1, 70) = 148.79, *p* < 0.001
Testosterone: Pre Post % change Significance (paired) *	0.65 (0.56–0.74) 0.27 (0.19–0.31) −58.5 *p* ≤ 0.001	0.51 (0.28–0.72) 0.26 (0.20–0.33) −49.0 *p* ≤ 0.001	*p* ≤ 0.66 *p* ≤ 0.001	Group Effect: F (1, 70) = 75.31, *p* < 0.001 Time Effect: F (1, 70) = 305.66, *p* < 0.001 Group × Time Interaction: F (1, 70) = 39.48, *p* < 0.001
LH: Pre Post % change Significance (paired) *	4.0 (2–10) 4.0 (2–10) 0 *p* ≤ 0.29	6.0 (3–10) 6.0 (3–9) 0 *p* ≤ 0.97	*p* ≤ 0.6 *p* ≤ 0.2	Group Effect: F (1, 70) = 6.48, *p* = 0.013 Time Effect: F (1, 70) = 1.93, *p* = 0.169 Group × Time Interaction: F (1, 70) = 3.7, *p* = 0.056
FSH: Pre Post % change Significance (paired) *	6.38 (5–7) 6 (4–6) −6.0 *p* ≤ 0.001	6.5 (5–7) 6 (4–6) −7.7 *p* ≤ 0.001	*p* ≤ 0.39 *p* ≤ 0.02	Group Effect: F (1, 70) = 2.86, *p* = 0.095 Time Effect: F (1, 70) = 60.1, *p* < 0.001 Group × Time Interaction: F (1, 70) = 1.87, *p* = 0.176
Prolactin: Pre Post % change Significance (paired) *	10 (7–13) 9.5 (6–12) −5.0 *p* ≤ 0.001	12 (8–13) 11.75 (8–13) −2.1 *p* ≤ 0.001	*p* ≤ 0.02 *p* ≤ 0.001	Group Effect: F (1, 70) = 11.01, *p* = 0.0014 Time Effect: F (1, 70) = 223.43, *p* < 0.001 Group × Time Interaction: F (1, 70) = 17.46, *p* < 0.001

* Wilcoxon signed ranks test; ART—Aligned Rank Transform; LH—luteinizing hormone; FSH—Follicle-stimulating hormone.

## Data Availability

The datasets employed in this study can be obtained from the corresponding author upon reasonable request. However, certain data cannot be made publicly accessible due to privacy considerations.

## Abbreviations

The following abbreviations are used in this manuscript:

HIIT	High-intensity interval training
TRT	Traditional resistance training
FSH	Follicle-stimulating hormone
LH	luteinizing hormone
